# Perceptions about family-centred care among adult patients with chronic diseases at a general outpatient clinic in Nigeria

**DOI:** 10.4102/phcfm.v10i1.1739

**Published:** 2018-10-23

**Authors:** Kenneth Yakubu, Zelra Malan, Maria C. Colon-Gonzalez, Bob Mash

**Affiliations:** 1 Department of Family Medicine, University of Jos and Jos University Teaching Hospital, Nigeria; 2 Division of Family Medicine and Primary Care, Stellenbosch University, South Africa; 3 Department of Family and Preventive Medicine, University of Texas Rio Grande Valley School of Medicine, United States of America

## Abstract

**Background:**

Few studies in Africa have described patients’ perceptions about family-centred care (FCC).

**Aim:**

The aim of this study was to explore perceptions of FCC among patients with chronic diseases.

**Setting:**

The study was conducted at a general outpatient clinic (GOPC) in Jos, north-central Nigeria.

**Methods:**

We used a mixed-methods phenomenological study design and conducted structured and semi-structured interviews with 21 adult patients with chronic diseases at a general outpatient clinic in north-central Nigeria.

**Results:**

Patients described FCC using progressive levels of family engagement including the doctor inquiring about history of similar disease in the family, information sharing with family members and fostering of family ties. They described current family involvement in their care as either inquiring about their health, accompanying them to the clinic or offering material or social support and health advice. Also, patients considered the value of FCC based on how it meets information needs of the family, influences individual health behaviour and addresses family dynamics. Those who were literate and older than 50 years of age favoured FCC during history taking. Those who were literate, aged lesser than 50 years and had poor disease control showed preference for FCC during treatment decision-making.

**Conclusion:**

The acceptability of FCC is a complex synthesis of age, socio-economic status, literacy and disease outcomes. Patients older than 50 years, with good treatment outcomes, and those without formal education may need further education and counselling on this approach to care.

## Introduction

The global burden of chronic diseases is on the increase. By 2020, the estimated prevalence in all people will increase to 57%, accounting for three-quarters of deaths worldwide.^1^ To address the resultant morbidity and mortality, as well as improve the quality of life, there is a need for innovative health care delivery models.^2^ Supporting self-care through the chronic care model is one such innovation.^2^ However, its effectiveness may be limited by a poor understanding among health workers, of the complex cultural contexts involved in dealing with chronic patients, as well as a failure to recognise that family has a significant influence on individual health behaviour.^3^ Family members are inevitably part of a patient’s social network and cultural identity, and their influence can be either supportive or detrimental in terms of improving self-care.^4^ To ensure families are enabled to provide the right kind of support for self-care, there is a need to invite, support and guide their involvement in the care of patients with chronic diseases.^5,6^

Focusing on facilitators of self-care may be termed as being ‘patient-centred’ or ‘family-centred’.^7,8^ While patient-centred care (PCC) and family-centred care (FCC) have been used interchangeably or in combination, some have interpreted the former to mean ‘patient-focused care’.^9^ Although ‘patient-focused care’ recognises the value of the patient’s family, it focuses on the patient’s preferences and values during the consultation.^7^ In contrast, FCC has been described as an approach to care that considers the needs of the family as well as that of the patient.^9^ Furthermore, it has been defined as an approach to health care delivery which empowers the family as an ally in the care of an individual.^10^ When viewed as partners with the family doctor, patients and their families can participate in diagnosis and treatment decisions.^11^

Different frameworks have been used to describe how FCC can be implemented. One of such is a consultation process that involves partnership, shared decision-making and shared leadership.^6^ Another described self-care and family care during consultations.^4^ However, when direct care of patients is considered, Cole-Kelly et al. and McDaniel et al. have described practical ways of providing family-oriented consultations in the clinic as well as a simple approach to describing family involvement in the treatment decision-making process.^12,13^ The approach described by Cole-Kelly includes questions on family history of the same disease and the patient’s opinion on how the family can help address his or her health concerns.^12^ McDaniel’s description of family involvement ranges from minimal involvement of the patient’s family in the care process to providing family therapy for a dysfunctional family.^13^

Family-centred care has been acknowledged internationally, as an essential part of the chronic care model.^14,15,16,17,18,19^ Arguments in favour of FCC have centred on its benefits which include equity in health care delivery, patient safety and improved quality of care.^3,20^ Other described benefits include reduction of medical costs for both the patient and health care facility, improvement of patient satisfaction and adherence to clinical management plans.^21^

Compared to developed countries, there appears to be a dearth in the literature, describing the concept and practice of FCC in an African context. However, available studies have shown that routine family-oriented interviews may increase perceived family function of young persons who receive medical care at a family medicine clinic in Nigeria.^22^ A study from Malawi showed that because of the paucity of health workers, family members were often involved in providing hospital care for their patients.^23^ In Lesotho and Mozambique, studies have shown that parents and other family caregivers were often left out of the care process because of frequent communication difficulties with health workers who treat their patients.^24,25^ At the time of this research, a PubMed and Google Scholar search using the following MeSH terms or keywords: ‘sub-Saharan Africa’, ‘patient’s perceptions’, ‘family-centred care’ and ‘chronic diseases’ did not yield relevant results.

In view of the need to promote FCC within an African context, eliciting patients’ perceptions of FCC is arguably a necessary step that should precede adaptation and implementation. Such perceptions can help promote an understanding of the patient’s receptivity and preference for FCC in this setting. Therefore, the overall aim of this study was to explore the perceptions of FCC among patients with chronic diseases at a general outpatient clinic (GOPC) in Nigeria.

The objectives of this study were to: (1) elicit patients’ perceptions of the meaning of FCC, (2) explore current involvement of family members in patient care, (3) explore the possible value of FCC and (4) explore patients’ preferences in the delivery of FCC.

## Methods

### Study design

This was a mixed-methods phenomenological study (Qual > Quan)^26^ in which structured and semi-structured interviews were employed.

### Setting

The study was conducted at a GOPC in, Jos, north-central Nigeria. At this GOPC, a daily average of 250 patients are seen with primary or secondary health care needs. These include acute and chronic medical conditions.^27,28^ The lead author is an honorary consultant family physician and practises in this clinic. Except for one of the patients involved in this study, the author was unfamiliar with all the other patients.

### Study population and sampling strategy

The study population consisted of adult patients with chronic diseases receiving care at a GOPC in Jos. As patients waited at the triage area, the lead author inspected their medical records and then selected patients using a purposive sampling technique based on the inclusion and exclusion criteria. To ensure maximum variation, patients from different genders, ethnicity, religions, socio-economic background, literacy level and chronic diseases were included in the study population. A sample size of 16 individual interviews was proposed as recommended by Reid and Mash.^29^ However, recruitment and data collection continued until 21 interviews were conducted in order to achieve saturation sampling.^30^

### Inclusion criteria

The inclusion criteria were as follows:

- Patients aged 18 years and older.
- Patients who did not require emergency or inpatient care.
- Patients who had one or more chronic diseases that were either physical or psychiatric. For the purpose of this study, chronic diseases were defined as any disease expected to last beyond 12 months and required ongoing medical care.^31^

### Exclusion criteria

The exclusion criteria were as follows:

- Patients who had cognitive deficits (such as the elderly with dementia and Parkinson’s disease) as documented in their health records.
- Patients who refused to give consent.

### Data collection

The lead researcher (Kenneth Yakubu [K.Y.]) performed three interviews as part of a pilot test. The aim of the pilot test was to ascertain ease of recruitment into the study, comprehension of the interview questions, the need for modification of the interview guide and the average time required to conduct each interview. He then went ahead to conduct 21 interviews in the language preferred by the patients (either in English or Hausa). An interview guide (Appendix 1) was used and it contained structured (closed-ended) and semi-structured (open-ended) questions. For the latter, techniques such as reflective listening, elaboration and summaries were used.^29^

For the open-ended questions, the interview guide included the following topics:

- The meaning of FCC from the patient’s perspective.
- Current family involvement in delivery of care.
- The possible value of FCC.

For exploring patients’ preferences in the delivery of FCC, we used closed-ended questions to start the conversation and this was followed with open-ended questions needed to probe and clarify the patient’s context. We explored patient’s preferences for FCC in two parts of the consultation process. These parts include the history taking and evaluation process, and the treatment decision-making process. To explore patient’s preferences for FCC during the first part of the consultation, we referred to five standard family-oriented questions and asked them to choose what they will want their physician to ask them during a consultation.^12^ More than one preference were permissible. Patient’s preferences for FCC in the second part of the consultation process (i.e. treatment decision-making) were explored by asking them to choose one option from five possible levels of family involvement.^13^ As standard frameworks for the delivery of FCC exist,^10,12^ this approach to data collation was aimed at generating theories as to why patient’s preferences aligned (or did not align) with the options provided in these frameworks. Details of the specific questions and statements in the interview guide are provided in Appendix 1.

### Data analysis

For each semi-structured interview, responses were transcribed verbatim, and K.Y. checked for errors by comparing each sheet with the audio recording. Analysis was done using Atlas.ti 8.0.^32^ The framework approach to thematic analysis was used as follows:

- Familiarisation: K.Y. and Maria C. Colon-Gonzalez (M.C.C-G.) familiarised themselves with the data by reading each transcript independently.
- Construction of thematic framework: Three documents were randomly selected and open-coding was independently done by both researchers. The codebooks were combined, and they agreed on a thematic framework.
- Coding: The thematic framework was applied to the data as both researchers annotated each of the transcripts using separate project bundles on Atlas.ti. This did not hinder emergence of new codes where necessary. Each researcher kept an audit trail and had up to three rounds of coding for each transcribed document. After the 18th transcribed interview, no new theme emerged (data saturation point). Nonetheless, coding was completed for all 21 transcribed interviews. Thereafter, they compared their coded transcripts and ensured that consensus was achieved on all codes used.
- Charting: After merging both project bundles and getting the report of all quotes used, K.Y. brought together all the data for each code group into a separate document (chart).
- Mapping and interpretation: K.Y. then read each chart and interpreted the data by looking out for recurring units of meaning (themes) and associations between them.

Both researchers (K.Y. and M.C.C-G.) then reviewed for internal consistency by mapping codes to the original quotes in the transcripts and by referring to their audit trails.^33^ Data saturation was reached within the results, and hence, further interviews were not required. For the structured interviews, frequencies were used to describe the distribution of patient’s preference for each of the five family-oriented questions and five levels of family involvement in patient care.

### Ethical considerations

Informed consent was sought and obtained from all participants, and the research was conducted in line with the Helsinki Declaration. Selection of study participants was based on documented inclusion and exclusion criteria with no bias or favouritism. The study posed no risk to the participants as no tissue or blood samples were required and no drugs administered. The questions asked did not create undue stress or anxiety in the participants as opinions about meaning, importance and preference for the delivery of FCC were elicited in a neutral, sensitive and respectful manner. Each participant was assigned an identification number, and only this number was stored; the identity of the participants was not revealed to ensure confidentiality.

IRB approval for the research protocol was obtained from the Health Research Ethics Committee (HREC) at Stellenbosch University (reference number: S16/07/133).

## Results

Twenty-one patients were interviewed, which included 12 females and 9 males aged between 20 and 70 years. They all received care for chronic diseases including hypertension, diabetes, osteoarthritis, hyperthyroidism, presbyopia, low back pain, peptic ulcer disease, depression and somatisation disorders. The demographics of the respondents is summarised in Table 1.

**TABLE 1:** Baseline characteristics of the respondents.

Sociodemographic variables	Frequency	Percentage
**Gender**		
Male	9	43
Female	12	57
**Age**		
< 50 years	12	57
≥ 50 years	9	43
**Tribe**		
Indigenous to Plateau state	5	24
Non-indigenous	16	76
**Religion**		
Islam	13	62
Christianity	8	38
**Family or household types**		
Monogamous	3	14
Polygamous	5	24
Extended family	6	29
Lives alone	3	14
Widow or widower but lives with children	4	19
**Occupation**	1	5
Unemployed	13	62
Self-employed	4	19
Employee in the public sector	2	9
Employee in the private sector	2	9
Student	1	5
**Education**		
Formal education (i.e. any of primary, secondary or tertiary education)	15	71
Qur’anic education only	4	19
No formal education	2	10
**Average monthly income**		
Less than N18 500	11	52
Between N18 500 and N85 000	8	38
More than N85 000	2	10
**Diagnosis**		
Physical or organic illness treated – controlled	13	62
Physical or organic illness treated – not controlled	5	24
Mental or non-organic illness treated – controlled	2	9
Mental or non-organic illness treated – not controlled	-	-
Organic and mental illness treated – not controlled	1	5

Note: monogamous, a husband and one wife; polygamous, a husband with more than one wife; extended family, either monogamous or polygamous but lives with other relatives of either the husband or the wife; Qur’anic education, a system of schooling focused on imparting students with knowledge from the Qur’an.

A summary of the themes has been provided in Table 2. The resultant themes and sub-themes have also been organised under each of the four study objectives posed as numbered items below. The age of the patients, type of household and education are written after each quote.

**TABLE 2:** Summary of the key themes.

Level of engagement with family	Meaning of FCC as perceived by the patients	Current involvement of family members in patient care	Value of FCC as perceived by the patients	Patients’ preferences for FCC
Low	Get information on family history of disease.	Family members ask questions about illness at home.	Informing family members can help prevent illness in the household.	Almost all appreciated the need to explore underlying genetic factors or stressors in the family Some preferred the doctor to plan treatment with just the patient or only involve family members for practical or legal reasons.
Moderate	Share information on patient’s illness with accompanying family members.	Family members accompany patient and ask their own questions.	Family members understand more about the illness and can therefore offer appropriate support.	Most appreciated the doctor exploring how the family could help and who was most supportive. A few wanted the doctor to address questions coming from the family about the treatment, but no one wanted the doctor to elicit the family’s feelings and concerns on this issue.
High	Health care fosters family relationships and cares for the family not just the patient.	Family members provide material (financial) support, social support, advice and encourage adherence to treatment.	FCC can explore the effect of the family dynamics on the illness.	About half appreciated the doctor exploring the family’s beliefs and opinions on the cause of the illness. Some wanted the doctor to explore how the family relationships might be contributing to the illness.

FCC, family-centred care.

### The meaning of family-centred care

Patients described it as the doctor getting to know the family history of a patient, showing love and concern for the family and their future, as well as allowing family members to accompany the patient during the care process:

‘You are supposed to know the history of my family, [*and the*] family history of diseases.’ (20 years old, extended family, formal education)

‘It means the doctor shows love and concern to that family, and for the future of that family that is why he is treating the patient in a family way.’ (56 years old, immediate family-monogamous, formal education)

‘…. they bring you to the hospital and they come and stay with you …’ (60 years old, widow who lives with her two sons, no formal education)

Patients thought that FCC also refers to how information about their illness and required treatment is shared with family members:

‘This type of seeing patient does not observe confidentiality, it allows family members to be part of the treatment.’ (55 years old, immediate-polygamous family, formal education)

‘Especially my parents, everything about my health, they should know.’ (33 years old, immediate family monogamous, formal education)

Patients considered this type of care as one which fosters family ties and builds relationships, which includes the doctor as an integral part of the family:

‘Like I told you at first, family, your life with them helps your own life especially the ones you know are your own, your blood.’ (30 years old, extended family, formal education)

‘It’s like having a relationship. So, the relationship with me and my family should be such that we become one. You and my family become ‘one broom’. (56 years old, widow who lives with her son, no formal education)

Furthermore, patients clarified the context within which FCC should be offered. They explained that this approach should not be a constant routine, but could be useful in instances of severe illness:

‘Your family, if the illness is severe they bring you to the hospital and they come and stay with you and be part of what is happening.’ (60 years old, widow who lives with her two sons, no formal education)

‘In the African setting…only when it’s severe, that’s when the family gets involved but if it’s not, you [*seek care*] alone.’ (38 years old, monogamous family setting, formal education)

### Family members’ involvement in the care of patients

Patients considered family members as being involved in their care when they came with them to the clinic. However, for some, this was an occasional practice and not the norm. Although patients valued being accompanied, they also reported that family members could not always accompany them:

‘Sometimes they come with me to the doctor’s office …’ (55 years old, extended family, formal education)

‘… nobody comes [*with me*] because they are young and my wife is at home taking care of the children.’ (39 years old, extended family, no formal education)

When questioned on how they felt about visiting the health facility unaccompanied, some patients explained that they did not consider being accompanied by family members to the clinic as their involvement in the care process, either because they were used to visiting the health facilities all by themselves or because they viewed their illness as not so severe to need help:

‘I am the only one who comes to the clinic because my illness is not severe.’ (widow, 60 years old, Qur’anic education)

‘I don’t think it is important because I have been coming here all alone.’ (33 years old, single, lives alone, formal education)

The patients felt that family members were also involved in their care by inquiring about their health and by asking about the care that they received at the hospital when they returned home. This made them feel cared for, even when these inquiries were mere formalities:

‘When I get back he asks me- how far? [*meaning how did it go?*] Did you see the doctor?’ ‘What happened?’ (38 years old, extended family, formal education).

‘They ask me questions because there’s nothing else they can do.’ (56 years old, widow but lives with children, no formal education)

Offering material and social support was another way for family members to be involved in a patient’s care. This ranged from offering money for hospital expenses and food, to assisting them with chores, and providing physical company and emotional support:

‘They help me a lot in coming here. They help me by giving me money to come to the clinic.’ (30 years old, single, lives alone but interacts with extended family, formal education)

‘He is the one that went around, did everything and they always stay by my [*side*].’ (56 years old, immediate-monogamous family, formal education)

‘She always motivates me …’ (37 years old, lives alone but interacts with immediate and extended family, formal education)

Family members were involved in patient’s care when they addressed health-seeking behaviour of the patients by offering advice on their health problems and also by encouraging the patients to adhere to management plans:

‘Some people were saying go and take this type and that type of medicine, maybe you will get better. My husband and children said no, … I should go and see the doctor, listen to what the doctor will evaluate …’ (56 years old, immediate-polygamous family, formal education)

‘She also ensures that I come to the hospital to get my check-ups.’ (28 years, immediate-monogamous family, formal education)

Some patients could not describe family members as being involved in their care. Others were either not dependent on the family for financial or other resources, or they could decide the extent to which family members were part of the care process because they were independent:

‘When I was well, my husband used to pay attention to me but now that I am ill, not even the blessing of my children makes him care for me …’ (36 years old, immediate – polygamous family, formal education)

‘Sometimes they come with me into the doctor’s office, other times I ask them to wait outside.’ (55 years old, polygamous family, formal education)

‘My family knows I am coming here but it’s not that they have been giving me anything so I can come here. It’s me that is looking for it by myself.’ (49 years old, widow but lives with her children, formal education)

### The value of family-centred care

Patients thought that FCC could address prevailing family dynamics and its influence on the patient’s illness experience. Furthermore, FCC could help improve their adherence to treatment and health outcomes:

‘If you take care of the family, in some instances you have taken care of the illness.’ (49 years old, immediate monogamous family, formal education)

‘Like I told you, it will help in healing someone through how he/she thinks. How he/she sees things … most relationships, especially the relationship we have at home with family, brings problems through thoughts.’ (30 years old, extended family setting, formal education)

‘She also ensures that I come to the hospital to get my check-ups.’ (28 years old, immediate monogamous setting, formal education)

Patients revealed that informing family members about their condition made them more willing and able to care for them:

‘Yes, so that they can care for me, like feeding, good food, the type of care that they will offer to me at home, knowing how my health is.’ (55 years old, immediate family, polygamous, no formal education)

‘Because if you are coming alone, one day when you explain it at home, they will say it’s a lie, it’s not like that, you just want them to give you money for you to use it.’ (30 years old, lives alone but interacts with extended family, formal education)

### Preferences of patients in the delivery of family-centred care

Table 3 shows the number of patients who responded positively to each question on patient’s preferences during the history taking and evaluation process. Even though the value of family involvement in an individual’s care was described by the patients, preference for any of the five family-oriented questions depended on whether confidentiality was a primary concern for the patient:

‘Yes he may ask if the illness requires it but I will not prefer it because it is a secret …’ (20 years old, extended family, with formal education)

‘No, there’s no breach of confidentiality with sickness.’ (36 years old, immediate family, polygamous with no formal education)

**TABLE 3:** Distribution of patient’s preferences to each of the family-oriented questions used during the consultation process.

Family-oriented questions during consultation	Frequency of participants who showed preference for each question
Would you want to be asked about similar health issues in your family?	20
Would you want to be asked about what your family members believed caused the problem?	11
Would you want to be asked who in your family is most concerned about your health?	15
Would you want to be asked about stressors or events in the family that may be contributing to your health issue?	17
Would you want your opinion sought on how your family can be helpful in addressing your health concern?	13

Note: Some patients showed a preference for more than one option, while others abstained from making a choice on any.

It also depended on whether the patient thought these questions were relevant in helping the family doctor know and/or understand more about their health:

‘If I express my opinion and they theirs, you [referring to the doctor] can understand better what I have not mentioned.’ (56 years old, immediate family polygamous, formal education)

‘There are benefits of asking … because there may be things that they may have seen that I did not see.’ (55 years old, immediate family, polygamous, formal education)

Not every patient thought that their satisfaction with care would increase when offered FCC, only those who wanted this type of care stated that it would:

‘I would be happy if he asks about my opinion because it shows he considers me to be important.’ (36 years old, immediate polygamous family setting, formal education)

‘I won’t be comfortable if the doctor asks me for my family’s opinion, -not at all. How would you expect my husband to know what caused the pain?’ (38 years old, immediate, monogamous family setting, formal education)

Preference for family involvement also depended on whose opinion mattered to the patient:

‘The opinion that matter is the doctor’s. If I had my opinion or my husband’s or my children’s or relatives, I won’t bring myself to the hospital.’ (70 years old, immediate family, monogamous; Qur’anic education only)

‘It is my opinion, it’s me that is ill, I will be the one to say how I am feeling and the condition.’ (60 years old, widow but lives with children and grandchildren, Qur’anic education only)

‘It’s better both sets of opinions are combined.’ (30 years old, lives alone but interacts with extended family, formal education)

The patients’ perception of the cause of their illness influenced their preferences. If the illness is linked to social problems in the family, then their involvement is necessary. However, if it has no clear origin, then patients will not want to involve their family members:

‘… there is a type of illness that is not God that brought it. It’s as result of problems … If you ask me today, you’ll understand that this is not really [*about*] severity of the illness itself, but the problems I have, that’s what increased my illness.’ (56 years old, widow lives with her children, no formal education)

‘I don’t think it is necessary for the doctor to ask me what they think because most of this sickness just come sometimes and you cannot say, this is the cause.*’* (33 years old, lives alone but interacts with immediate family, formal education)

Patients wanted to involve family members if they thought that it could provide an entry point for them to receive health care themselves:

‘I will be very happy [*if I am asked*] because knowing if there is a similar case in my family, it may help to improve their health. You may even wish to invite the persons.’ (37 years old, lives alone, formal education)

‘If they express their own opinion it is possible that they have an illness that I don’t know about. That can be an opportunity for them to say: I have such and such a problem.’ (56 years old, immediate polygamous family setting, formal education)

The patients had different preferences on the extent to which family members could be involved during the treatment decision-making process, but most of them showed a preference for either a low level of family engagement (denoting a patient-focused decision-making process, Options 1 and 2, Table 4) or a high level of family engagement (denoting a family-centred decision-making process, Options 3 and 5, Table 4). However, they did not show a preference for dealing with the possible emotional impact their disease or their treatment may have on the family.

**TABLE 4:** Patient’s preferences for the delivery of family-centred care during treatment decision-making.

Treatment decision-making option	*N*
The doctor focuses only on what you want and expects that your family members will respect your wishes.	4
The doctor contacts your family only when there are practical or legal reasons.	4
The doctor communicates with your family about your treatment plan, addresses any practical question they may have and agrees with them, on action plans.	3
The doctor’s involvement goes beyond practical questions and allows your family to express their feelings and concerns about the treatment plan and shows them empathy.	0
The doctor assesses the connection between your illness and relationship within your family, as well as works with the family to resolve it.	5
None of the above.	5

Similar to preferences during history taking, some patients were concerned about confidentiality. For others, there was a preference for a ‘patient–doctor’ dyad alone because of personal information they wished to keep secret. Yet, others thought sharing these secrets with a spouse helped to foster trust between them. Instead of focusing on medical issues only, some considered a ‘patient–family’ dyad when it concerned financial implications of their care:

‘… there are somethings I can tell the doctor, I and him, but I can’t tell my brothers.’ (55 years old, polygamous family setting, formal education)

‘It will improve trust between us since this means that I trust her so much to the extent that I can allow her see all my problems and allow her come to the hospital with me.’ (28 years old, monogamous family setting, formal education)

‘… everything they are supposed to know but when it comes to money, its personal, allow me and my family talk about it.’ (20 years old, extended family setting, formal education)

The patients’ preference was influenced by their perception of the illness experience. It depended on whether they thought their illness was severe or not, and if they saw it as an individual experience (i.e. the more severe the illness, the more value in involving the family). If family members could benefit from knowledge about the disease, and thus prevent the illness occurring in other family members, it was seen as worth sharing with the family:

‘If the illness becomes very severe and you need to be admitted, this will involve the whole family.’ (28 years old, monogamous family setting, formal education)

‘I prefer he stays with my opinion because I am the one who is going through the illness.’ (60 years old, polygamous family setting, no formal education)

‘It could be from what you eat or drink, so for participation of the family, they will benefit from getting advice that can prevent illness.’ (39, extended family setting, formal education)

Patients had preferences for different power sharing models during decision-making. Some indicated their preference for either their parents or the doctor to have more powers in the decision-making process. Others showed preference for equal decision-making powers involving the patient and the family:

‘The opinion that matters is the doctor’s.’ (70 years, monogamous family setting, Qur’anic education only)

‘It’s them, the parents that should be involved. It’s them that should even be at the fore front of decision-making.’ (30 years, monogamous family setting, formal education)

‘I prefer he will hear the complaint from me then invite my family to learn their opinion.’(Female, 36 years, formal education)

Interestingly, the patients did not think home visits were necessary for making decisions about their treatment:

‘… even if I am not visited at home, if my children are in town …no one will refuse to come [*to the clinic*]. And anyone that will come, will contribute their own opinion when asked.’ (56 years old, polygamous family setting, formal education)

‘Anyone the doctor does is okay but as for me, I do not have a problem at home that will require that you to meet with people at my home.’ (60 years old, widow but lives with children and grandchildren, Qur’anic education only)

Table 5 shows the distribution of preferences for FCC across the various subgroups.

**TABLE 5:** Subgroup distribution of preferences for family-centred care during history and treatment decision-making.

Family-oriented questions during history taking	Subgroups that showed preference for this	Treatment decision-making options	Subgroups that showed preference for this
Question 1	†	Option 1	High-income group, good disease control, > 50 years
Question 2	> 50 years,	Options 2	†
Question 3	Formal education, > 50 years	Options 3 or 5	Formal education, poor disease control, <50 years
Question 4	†	Options 4	†
Question 5	Formal education	-	-

† , No distinct difference across the groups.

## Discussion

We found that the respondents had a broad range of preferences concerning FCC ranging from a low to high level of engagement with the family, consistent with other studies which have also showed preferences ranging from minimal to maximal family involvement in care.^34,35^ A distinct preference for family involvement during history taking was seen for those aged greater than 50 years and those who had formal education. For treatment decision-making, a high level of family engagement was favoured when the illness was severe or poorly controlled, when the patient was aged less than 50 years and had formal education, when there was little need for confidentiality, when the cause was believed to involve the family (e.g. genetic diseases or stressors from family relationships) and when practical or financial support was required. Other factors that appear to influence the individual’s preference for the various levels of engagement include income level of patient, perceived value of FCC, need for confidentiality and family support.

There appears to be a complex relationship between age of the patient and preference for FCC. While other studies have found that family involvement in the care of elderly patients was desired,^36^ ours seem to suggest that this preference is limited to history taking. Low educational level has been found to be a predictor for family involvement in care.^37^ However, this might be family members’ preference to protect their patients from the complexity of health care systems. It is possible that respondents who were literate in our study were left to navigate care by themselves, hence their desire for support and company.

Concerning the value of FCC, most patients expected to receive either or all of social, material, emotional and financial support from their family. This suggests a reluctance to be isolated from the family system and is consistent with descriptions of communal living that characterises the African experience.^38^ However, family interaction can produce conflicts which may be a source of social stress.^39^ Hence, in addition to factors already mentioned, dealing with such family conflicts might be an additional factor that explains why some patients preferred the involvement of one supportive family member compared to the whole family unit, as well as why others preferred minimal family involvement instead of a maximum family engagement.

Furthermore, if the value of health care is seen as clinical outcomes relative to cost,^40^ then this study’s findings on the value of FCC, particularly about empowering the family and improved adherence, are consistent with what has already been described as the benefits of FCC.^3,20,21^ However, it is interesting to note that even though FCC can ultimately lead to appropriate use of resources and reduction in cost of care,^9^ our patients did not specifically mention this. As responses in this study are limited to the patient’s perceptions, future research should focus on the economic benefit of FCC.

Our findings on current family involvement in patient care still show that the family plays a key role in the health delivery process of patients. For most patients, this study showed that the family was their main source for material and financial support. This is important, because most health payment options in Nigeria are out-of-pocket.^41^ While this supports further argument for a perceived value for FCC in this setting, it raises concerns about catastrophic health expenditure for families with limited resources, faced with a lifetime commitment to caring for their members with chronic diseases.^42^

The patients showed that they had a good understanding of the meaning of FCC. Their perspective on the meaning of FCC is consistent with current principles.^9^ These principles include information sharing, honouring and respecting differences, partnership or collaboration and care in the context of the family or community.^9^ While this suggests that FCC is well understood, our study did not show that all patients preferred maximum family engagement during their care. Rather, our findings demonstrate that in addition to understanding the meaning of FCC, perceived value of FCC, high-income status, low literacy levels, patient autonomy, age and the need for confidentiality are possible barriers to its receptivity among patients.

### Limitations

The pattern of emergent themes in our study and the quantitative description of patient’s preference may not be reflective of its natural occurrence in the study population. Despite these limitations, our study elicits an understanding of patients’ preferences and could therefore offer a guide to the practice of FCC for family doctors in this setting.

### Recommendations

Considering the range of preferences for FCC, we recommend that the family doctor begins the consultation process with less sensitive family-oriented questions (i.e. history of similar disease in the family, significant events and identifying who the significant other is) and only proceeds to the other family-oriented questions if the patient is receptive. While elderly patients may prefer family involvement during history taking and evaluation, the family doctor should be sensitive to their need for autonomy during decision-making. Also, even though patients who are literate can navigate health delivery by themselves, their need for family support should not be ignored. For busy clinics, the nurse can help elicit patient’s preference for FCC and indicate this on the patient’s medical records. In addition, health service managers should make the consultation rooms more conducive for family members as well as provide a room for family conferencing, should it be necessary to interact with a whole family unit. There is a need for studies aimed at determining the feasibility of routine FCC provision in busy outpatient clinics, the economic implications for the patient and the health care provider, the distribution of these preferences in a representative population as well as elicit preferences for FCC among family members of patients with chronic diseases. Physician readiness and preferences among other health workers will also need to be assessed.

## Conclusion

By eliciting patients’ perceptions on the meaning of FCC, its value and current family involvement in their care, our study suggests that a range of preferences exists at a GOPC in a Nigerian setting. Hence, in promoting the uptake of FCC, paying attention to potential barriers may help in the prompt identification of patients who may need further education and counselling on the relevance of FCC to their care.

## Appendix 1

### Section C: Interview guide

#### Welcome and overview:

Hello, I am Dr Yakubu and I will like to interview you about family centered care.

**1. Read & review participant informational script.**

**2. Ground rules for the session:**

“There are no right or wrong answers to any of the questions that I will ask you today. As we discussed earlier in the informational script, I would like to audio record our session to make sure that I do not miss any important information by taking notes alone. Your name will not be used during the transcription process in order to protect your privacy. If you agree, please try to speak clearly and about as loud as I am speaking now. Thank you”

**3. Request and answer any questions.**

**4. Individual address:**

“In order to maintain your confidentiality, I will not be addressing you by your given name. How would you like me to address you (Options: Sir, Madam, Doctor, or Professor)? When the interview is complete, all audio data and notes will be identified only by a *study identification number* that is known to me alone.”

**5. Request and answer any questions**

“Are you ready to begin?”

**6. Begin recording** – *STATE the study identification number*_______, DATE, & TIME______

Ensure that participant is referred to as requested and NOT their given name.

**8. Key question:** “How do patients with chronic diseases perceive family-centred care?”

**9. Areas to explore**

#### I. Interviewee characteristics.

- Elicit and record baseline characteristic of the interviewee.

#### II. Participant’s opinion about involvement of family members in their treatment.

- How are your family members currently involved in your care?
- Probe on and clarify each opinion offered.

#### III. Meaning of family-centred care.

- What do you understand by care of a patient that is family-centred or focused?
- Probe on and clarify each opinion offered.

#### IV. Importance of family-centred care.

- What is your opinion about the value of family-centred care?
- Clarify that there are no right or wrong answers and FCC may be unimportant to the interviewee. Probe every point stated by interviewee.

#### V. Participant’s preferences in the delivery of family-centred care versus individual based care.

- When the doctor is asking you questions about your health concern; would you want him/her to:
  - Inquire about history of similar health issues in your family? (Probe why)
  - Ask questions about what your family members believed caused the problem
  - Ask who in your family is most concerned about your health? (Probe why)
  - Inquire about stressors or events in the family that may be contributing to your health issue? (Probe why)
  - Seek your opinion on how your family can be helpful in addressing your health concern? (Probe why).
- When a treatment decision is going to be made for you, which of the options below would you prefer: (for the one option selected, probe why).
  - The doctor focuses only on what you want and expects that your family members will respect your wishes.
  - He/she contacts your family only when there are practical or legal reasons. (e.g. of practical reasons is when you need in-hospital care and you need someone to stay with you).
  - He/she communicates with your family about your treatment plan, addresses any practical question / concern they may have and agree upon action plans.
  - As against (iii) above, he/she goes beyond practical questions, allows your family to express their feelings and concerns about the treatment plan and empathises with them.
  - Assesses the connection between your illness and relationship within your family as well as works with the family to resolve it.

**10. Closing question**

Do you have any additional comments or information?

**11. Summary**

- Summarise major comments for II – Vb
- Thank participant for the time spent.
- Follow up with a thank you note via sms on the same day.
